# The effect of exercise intensity and cardiorespiratory fitness on the kinetic response of middle cerebral artery blood velocity during exercise in healthy adults

**DOI:** 10.1152/japplphysiol.00862.2021

**Published:** 2022-06-16

**Authors:** Max E. Weston, Alan R. Barker, Owen W. Tomlinson, Jeff S. Coombes, Tom G. Bailey, Bert Bond

**Affiliations:** ^1^Children’s Health and Exercise Research Centre, Sport and Health Sciences, College of Life and Environmental Sciences, University of Exeter, Exeter, United Kingdom; ^2^Physiology and Ultrasound Laboratory in Science and Exercise, School of Human Movement and Nutrition Sciences, The University of Queensland, Brisbane, Australia; ^3^School of Nursing Midwifery and Social Work, The University of Queensland, Brisbane, Australia

**Keywords:** cerebral blood flow, exercise, kinetics

## Abstract

The aim of this study was to compare the kinetic response of middle cerebral artery blood velocity (MCAv) to moderate- and heavy-intensity cycling in adults, and explore the relationship between maximal oxygen uptake (V̇o_2max_) and MCAv kinetics. Seventeen healthy adults (23.8 ± 2.4 yr, 9 females) completed a ramp incremental test to exhaustion on a cycle ergometer to determine V̇o_2max_ and the gas exchange threshold (GET). Across six separate visits, participants completed three 6-min transitions at a moderate intensity (90% GET) and three at a heavy intensity (40% of the difference between GET and V̇o_2max_). Bilateral MCAv was measured using transcranial Doppler (TCD) ultrasonography and analyzed using a monoexponential model with a time delay. The time constant (τ) of the MCAv response was not different between moderate- and heavy-intensity cycling (25 ± 10 vs. 26 ± 8 s, *P* = 0.82), as was the time delay (29 ± 11 vs. 29 ± 10 s, *P* = 0.95). The amplitude of the exponential increase in MCAv from baseline was greater during heavy-intensity cycling (23.9 ± 10.0 cm·s^−1^, 34.1 ± 14.4%) compared with moderate-intensity cycling (12.7 ± 4.4 cm·s^−1^, 18.7 ± 7.5%; *P* < 0.01). Following the exponential increase, a greater fall in MCAv was observed during heavy-intensity exercise compared with moderate-intensity exercise (9.5 ± 6.9 vs. 2.8 ± 3.8 cm·s^−1^, *P* < 0.01). MCAv after 6 min of exercise remained elevated during heavy-intensity exercise compared with moderate-intensity exercise (85.2 ± 9.6 vs. 79.3 ± 7.7 cm·s^−1^, *P* ≤ 0.01). V̇o_2max_ was not correlated with MCAv τ or amplitude (*r* = 0.11–0.26, *P* > 0.05). These data suggest that the intensity of constant-work rate exercise influences the amplitude, but not time-based, response parameters of MCAv in healthy adults, and found no relationship between cardiorespiratory fitness and MCAv kinetics.

**NEW & NOTEWORTHY** This is the first study to model the MCAv kinetic response to moderate- and heavy-intensity cycling in healthy adults. This study found that the amplitude of the exponential rise in MCAv at exercise onset was greater during heavy-intensity exercise (∼34%) compared with moderate-intensity exercise (∼19%), but the time-based characteristics of the responses were similar between intensities. Higher cardiorespiratory fitness was not associated with a greater or faster MCAv response to moderate- or heavy-intensity exercise.

## Graphical abstract

**Figure:**
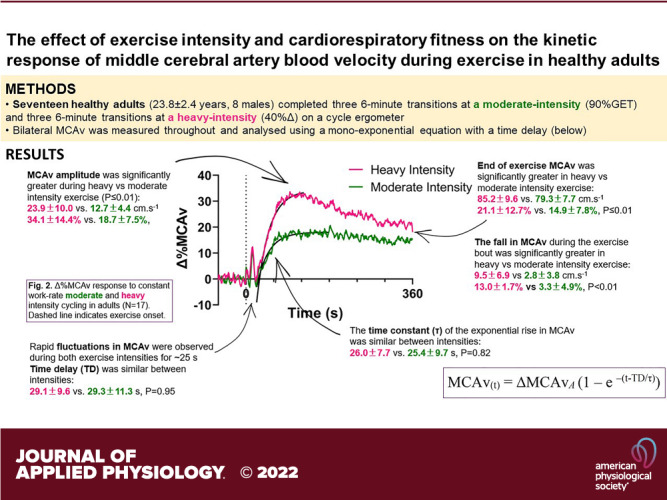

## INTRODUCTION

Studying the kinetic profiles of physiological variables from rest to constant work-rate exercise can provide valuable data on the time and amplitude-based characteristics of these responses (1). Furthermore, this approach can afford insight into control mechanisms during exercise, which is not possible by studying steady-state responses alone, by investigating how various physiological variables respond to exercise across time. A seminal study by Billinger et al. (2) found that the response of middle cerebral artery blood velocity (MCAv) to moderate-intensity exercise was well characterized by a monoexponential function with a time delay and has since been applied in the context of aging (3) and disease (4, 5). Ward et al. (3) observed a smaller MCAv amplitude and a slower dynamic response (greater time constant, τ) to moderate-intensity exercise in old compared with young adults. Furthermore, a blunted MCAv amplitude to moderate-intensity exercise has been observed in patients with stroke (2, 4). However, a limitation of these previous studies is the prescription of moderate exercise intensity using a percentage of predicted maximum heart rate (HR_max_; 2, 3, 6), which does not consider the location of an individual’s gas exchange threshold (GET; 7, 8).

When prescribing exercise intensity, it is important to consider the GET, as the physiological responses to exercise above and below the GET are distinctly different (9, 10). In particular, during moderate-intensity exercise (performed below the GET), steady-state responses of oxygen uptake (V̇o_2_), carbon dioxide production (V̇co_2_), minute ventilation (V̇e), end-tidal pressure of carbon dioxide ($\text{P}\text{E}{\text{T}}_{\text{C}{\text{O}}_{2}}$), and heart rate (HR) are observed (7). During heavy intensity exercise, performed above the GET and below critical power, hyperventilation occurs, alongside progressive increases in V̇o_2_, V̇co_2_, V̇e, and heart rate (11). These distinctly different physiological responses above and below the GET may be particularly important when studying cerebrovascular responses to exercise, given the close relationship between the cerebrovascular and respiratory systems (12).

Only one study has explored the effect of exercise intensity on MCAv kinetics. Witte et al. (6) observed a greater MCAv amplitude, but similar τ and time delay, during moderate-intensity stepping exercise (45%–55% heart rate reserve) compared with low-intensity (30%–40% heart rate reserve) stepping exercise. However, no study has explored the MCAv kinetic response to constant work-rate exercise greater than moderate intensity. Moderate-intensity exercise is considered to elicit the greatest increases in MCAv during exercise, due to the inverse parabolic response in MCAv seen during incremental exercise (13, 14). MCAv increases by ∼20%–30% in healthy adults up to 50%–60% of maximal work rate (W_max_), and then decreases with greater exercise intensity to values near, or below, the baseline at exhaustion (14, 15), predominantly as a result of hyperventilation-induced hypocapnia (15–17). It could, therefore, be hypothesized that the MCAv kinetic response to exercise above the GET may be more complex than during moderate-intensity exercise, possibly characterized by a fall in MCAv following an initial exponential-like increase.

As well as exercise intensity, cardiorespiratory fitness may also influence the cerebral blood flow (CBF) response to exercise. Witte et al. (6) observed a significant, positive correlation between estimated maximal oxygen uptake (V̇o_2max_) and MCAv amplitude during low (*r* = 0.41) and moderate (*r* = 0.50) intensity exercise in healthy adults. However, limitations in exercise intensity determination and V̇o_2max_ estimation through questionnaire compared with a direct measurement during an incremental exercise test to exhaustion (18), highlights the need for further investigation.

The purpose of this study was to compare the kinetics parameters of the MCAv response to moderate- and heavy-intensity cycling in healthy adults using an exponential model. Second, this study explored whether V̇o_2max_ was associated with MCAv kinetic responses to moderate- and heavy-intensity cycling. It was hypothesized that: *1*) MCAv would increase exponentially at the onset of moderate- and heavy-intensity cycling in healthy adults with similar time-based characteristics; *2*) moderate-intensity exercise would elicit greater increases in MCAv compared with heavy-intensity exercise, and MCAv would decrease as the heavy-intensity bout progressed; and *3*) a greater V̇o_2max_ would be associated with a greater MCAv amplitude during moderate- and heavy-intensity cycling in healthy adults.

## METHODS

### Participants

Seventeen healthy young adults (8 males and 9 females) participated in this study. Participants were recruited using convenience sampling and were postgraduate or undergraduate students from the University of Exeter. Following approval from the Sport and Health Sciences Ethics Committee, University of Exeter (190327/B/01), written informed consent was obtained for all participants. Participants were screened for the study exclusion criteria, which included contraindications to maximal exercise, current use of any supplement or medication is known to influence blood vessel function, smoking/vaping, and current or previous metabolic, cardiovascular, or cerebrovascular disease.

### Experimental Protocol

Participants visited the laboratory seven times. On the first visit, stature and body mass were measured following standard procedures, before participants completed a ramp incremental test to exhaustion on a cycle ergometer (Lode Excalibur, Lode, Groningen, The Netherlands).

#### Ramp incremental exercise.

Participants completed 3 min of seated rest on the cycle ergometer, before completing a ramp incremental test to exhaustion at a rate of 20–30 W·min^−1^. Participants were asked to maintain a consistent cadence between 70 and 90 revolutions/min (rpm) throughout the test. Exhaustion was deemed to have been reached when the cadence fell below 70 rpm for five consecutive seconds, despite strong verbal encouragement from the researchers. After 10 min of rest on the ergometer, participants completed a supramaximal verification test, performed at 105% of their ramp test peak power, until exhaustion (19). Participants wore a leak-free facemask (Hans-Rudolph, KS) connected to a metabolic cart (Medgraphics Cardiorespiratory Diagnostics, UK). Breath-by-breath pulmonary oxygen uptake (V̇o_2_), carbon dioxide production (V̇co_2_), and minute ventilation (V̇e) were collected and exported as 10 s stationary averages. V̇o_2max_ was defined as the highest 10 s averaged V̇o_2_ achieved during the ramp test or the verification bout (19). In 14 participants (82%), V̇o_2max_ was achieved during the ramp incremental test, whereas three participants achieved a higher V̇o_2_ during the supramaximal test. The V̇o_2_ corresponding to the GET was determined as the disproportionate increase in V̇co_2_ relative to V̇o_2_ (20) during the ramp test, and verified by an increase in the ventilatory equivalent of oxygen (V̇e/V̇o_2_) without an increase in the ventilatory equivalent of carbon dioxide (V̇e/V̇co_2_).

### Experimental Visits

Participants then visited the laboratory six times at the same time of day (±1 h), with ≥24 h between visits. All visits were completed in a mean of 20 ± 6 days (11–31 days). Participants arrived following a ≥2 h fast and having avoided caffeine (21), alcohol (22), and vigorous exercise (23) for the 24 h preceding the visit. On each of these six visits, participants completed either a bout of moderate-intensity cycling (performed at a V̇o_2_ corresponding to 90% GET) or a bout of heavy-intensity cycling (performed at a V̇o_2_ corresponding to 40% of the difference between GET and V̇o_2max_, 40%Δ; 7). The work-rates corresponding to these V̇o_2_ values were obtained from the linear relationship between work rate and V̇o_2_ during the ramp test and adjusted for a 30 s mean response time (MRT; 24). In total, participants completed three bouts of moderate and three bouts of heavy intensity cycling, performed in a counterbalanced order. Each bout consisted of a 3 min, seated, stationary baseline on the cycle ergometer, before an instantaneous transition to the target work rate. Participants cycled for 6 min and maintained a consistent cadence between 70 and 90 rpm.

### Experimental Measures

MCAv was measured bilaterally in all participants on every visit using transcranial Doppler (TCD) ultrasonography (DWL, Compumedics, Germany). Insonation of the left and right MCA was performed from an initial depth of 45–50 mm using two 2 MHz probes, secured in place with an adjustable headset (DiaMon, DWL, Germany). The position and depth of the probe were recorded for each participant and replicated between days. MCAv and HR data were collected at 200 Hz using an analog-to-digital converter (Powerlab; model: 8/30, ADInstruments) interfaced with a laptop computer and stored for off-line analysis (LabChart 8, ADInstruments). Breath-by-breath V̇o_2_ and V̇e data were also collected throughout (Medgraphics Cardiorespiratory Diagnostics, UK).

### Data Analyses

Mean MCAv data were exported as 1 s averages and time aligned to exercise onset. To create a single MCAv response for each intensity, for each participant, left and right MCAv data were averaged for each bout, and then ensemble-averaged with the corresponding repeat transitions to enhance the signal-to-noise ratio of acquired data. Each MCAv response was baseline-corrected for the 60 s preceding exercise onset, which was analyzed using a monoexponential model with a time delay (Eq. *1*) using GraphPad Prism (Graphpad Software, San Diego, CA).

(*1*) $$\text{MCAv}\left(t\right)=\;\Delta {\text{MCAv}}_{\text{A}}\left(\text{1 }-{\text{ e}}^{-(t-\text{TD}/\tau )}\right),$$

where MCAv(*t*) is the MCAv at a given time (*t*), ΔMCAv_A_ is the amplitude change of MCAv from baseline to its asymptote, TD is the time delay, and τ is the time constant.

**Figure 1.**
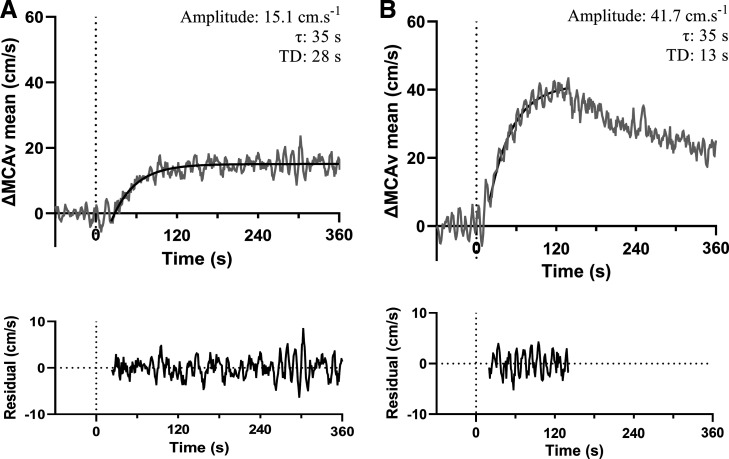
A representative trace for one participant for moderate (*A*) and heavy (*B*) intensity cycling. Data shown as an average of left and right MCAv responses across three repeat transitions, with the residual plot included below each figure. Time 0 s indicates onset of exercise. Data show a clear delay from exercise onset before MCAv increased exponentially. In *A*, MCAv attained a steady state, and the monoexponential was fit to the end of exercise (360 s). In *B*, a clear decrease in MCAv seen from *t* = 141 s, representing the end of the model fit. MCAv, middle cerebral artery blood velocity; τ, time constant; TD, time delay.

During both moderate- and heavy-intensity exercise, dynamic fluctuations in MCAv were observed for ∼20–25 s following exercise onset, before an exponential-like increase in MCAv was observed (see Fig. 1). The majority of MCAv responses to both intensities did not achieve or maintain a steady state, with MCAv often decreasing as the exercise bout progressed following the initial exponential rise (see Fig. 1*B*). Therefore, to improve and optimize the fit of the exponential model, MCAv data were modeled from the start of the exponential rise until departure from an initial steady-state amplitude. The start, end, and overall fit of each exponential model were verified by three researchers (BB, AB, and OT). To determine the appropriateness of each model fit, the residuals of each fit were inspected (Fig. 1), and the standard error of the τ was extracted.

Baseline MCAv was taken as the 60 s of seated rest preceding exercise onset. The amplitude of the exponential increase in MCAv was expressed as an absolute (cm/s) and relative (Δ%) change from baseline, as was MCAv at the end of the exercise bout, taken as the average of the last 10 s. The mean response time (MRT) of the MCAv response was calculated as the sum of the τ and TD.

To explore the test-retest reproducibility of kinetics parameters, left and right MCAv data were averaged for each transition separately, resulting in three moderate and three heavy MCAv responses for each participant. These were then separately modeled using Eq. *1*.

### Statistical Analysis

All data are presented as means ± standard deviation (SD). Statistical analyses were performed using SPSS version 26 (IBM), with statistical significance set a priori at an α-level of 0.05. Before completing the data analysis, a two-way mixed model analysis of variance (ANOVA) was used to investigate if there was an effect of sex on the MCAv response to exercise. No main effect of sex was observed for any kinetic parameter (*P* = 0.16–0.95, η_p_^2^ = 0.00–0.13), therefore male and female data have been combined and data are presented for the whole sample (*n* = 17).

Differences in kinetics parameters between moderate- and heavy-intensity exercise were explored using paired samples *t* tests. Effect sizes have been calculated and reported to support the use of the *P* value. An effect size (*d*) was interpreted as small if <0.5, moderate if 0.5–0.79, and large if ≥0.8 (25).

The reproducibility of kinetics parameters was explored using typical error, expressed as a coefficient of variation and intraclass correlation coefficient (ICC; 26). The relationship between V̇o_2max_ and MCAv amplitude and τ were explored using Pearson’s correlation.

V̇o_2_, V̇e, and HR data were exported as 1 s averages, time aligned to exercise onset and ensemble-averaged for each intensity, with end-exercise values taken from the last 10 s of exercise.

## RESULTS

Participant descriptive characteristics and responses to the ramp incremental test are shown in Table 1. Participants verbally confirmed they had adhered to the previsit instructions before each laboratory visit.

**Table 1.** Participant characteristics and incremental ramp test responses

	*n* = 17 (9 Female)
Age, yr	23.8 ± 2.4
Stature, cm	173.0 ± 9.3
Body weight, kg	70.9 ± 13.3
V̇o_2max_, L·min^−1^	2.71 ± 0.64
V̇o_2max_, mL·kg·min^−1^	39 ± 9
GET, L·min^−1^	1.28 ± 0.31
GET, %V̇o_2max_	48 ± 6
Moderate intensity work rate, W	80 ± 22
Heavy intensity work rate, W	159 ± 35

Moderate-intensity exercise performed at a work rate corresponding to 90% GET. Heavy-intensity exercise performed at a work rate corresponding to 40%Δ, where Δ is the difference between GET and V̇o_2max_. GET, gas exchange threshold; V̇o_2max_, maximal oxygen uptake.

The MCAv response to the onset of both moderate- and heavy-intensity exercise was well fitted by a monoexponential model with a time delay in all participants. This was shown by appropriate levels of test-retest reproducibility of kinetics parameters, and the τ standard error was 2.8 ± 1.1 s (range: 1.5–5.9 s) and 2.4 ± 1.4 s (range: 1.0–7.1 s) for moderate- and heavy-intensity exercise, respectively.

### The Effect of Exercise Intensity on MCAv Kinetics

**Figure 2.**
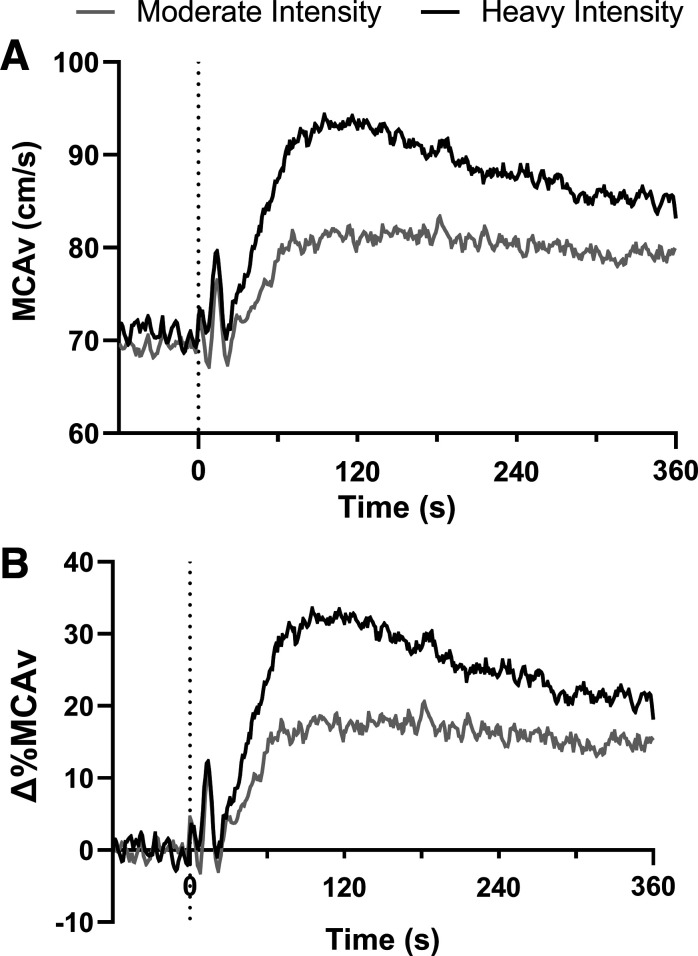
Group-averaged absolute (*A*) and relative (*B*) middle cerebral artery blood velocity (MCAv) responses to moderate (gray line) and heavy (black line) intensity cycling in healthy adults (*n* = 17, 9 females). Dashed line indicates exercise onset. Δ%, relative change from baseline.

The MCAv response to moderate- and heavy-intensity exercise is shown in Fig. 2. MCAv kinetics parameters for moderate- and heavy-intensity cycling are shown in Table 2. Baseline MCAv before each trial was similar between intensities. The time delay, τ, and MRT were similar between moderate- and heavy-intensity exercise. The amplitude of the exponential increase in MCAv was greater during heavy-intensity exercise, compared with moderate-intensity exercise, both in absolute and relative terms. After attaining a peak or steady state during the initial exponential rise, MCAv decreased in most participants during both moderate- and heavy-intensity exercise. MCAv maintained a steady state in only four adults (all during moderate-intensity exercise), and only in these cases were the exponential model fit to the end of exercise (*t* = 360 s). The decrease in MCAv occurred earlier and was of a greater magnitude during heavy-intensity exercise compared with moderate-intensity exercise. Nevertheless, MCAv at the end of the exercise bout was greater during heavy-intensity exercise compared with moderate-intensity exercise.

**Table 2.** MCAv kinetics parameters to moderate- and heavy-intensity cycling in healthy adults

	Moderate Intensity	Heavy Intensity	*P*	*d*
Baseline MCAv, cm·s^−1^	69.4 ± 9.1	70.8 ± 8.4	0.16	0.2
MCAv τ, s	25.4 ± 9.7	26.0 ± 7.7	0.82	0.1
MCAv amplitude, cm·s^−1^	12.7 ± 4.4	23.9 ± 10.0	**<0.01**	1.5
MCAv amplitude, %	18.7 ± 7.5	34.1 ± 14.4	**<0.01**	1.3
MCAv TD, s	29.3 ± 11.3	29.1 ± 9.6	0.95	0.0
MCAv MRT, s	54.7 ± 11.6	55.1 ± 11.8	0.88	<0.1
MCAv_end_, cm·s^−1^	79.3 ± 7.7	85.2 ± 9.6	**<0.01**	0.7
MCAv_end_, %	14.9 ± 7.8	21.1 ± 12.7	**0.01**	0.6
Onset of fall in MCAv, s	248 ± 90	164 ± 43	**0.01**	1.2
Fall in MCAv, cm·s^−1^	9.5 ± 6.9	2.8 ± 3.8	**<0.01**	1.2

Data shown as means ± standard deviation; *n* = 17 subjects. Bold indicates significant differences. *d*, Effect size; MCAv, middle cerebral artery blood velocity; MCAv_end_, MCAv at end of exercise bout; MRT, mean response time; τ, time constant; TD, time delay.

### Test-Retest Reproducibility of Kinetics Parameters

Baseline MCAv showed high test-retest reproducibility for both moderate- and heavy-intensity exercise, with a coefficient of variation of 7.1% and 7.0% and an ICC of 0.78 and 0.75, respectively. Across all six experimental visits, baseline MCAv had a coefficient of variation of 7.2% and an ICC of 0.77. The test-retest reproducibility of all kinetics parameters during moderate- and heavy-intensity exercise are shown in Table 3.

**Table 3.** Test-retest reproducibility of MCAv kinetics parameters to moderate- and heavy-intensity cycling in healthy adults

	Moderate Intensity		Heavy Intensity	
	CV (%)	ICC	CV	ICC
MCAv τ, s	35.9	0.64	19.5	0.77
MCAv amplitude, cm·s^−1^	8.7	0.65	7.8	0.79
MCAv amplitude, %	45.6	0.55	20.3	0.86
MCAv TD, s	42.0	0.70	22.7	0.82
MCAv MRT, s	21.8	0.55	11.8	0.78
MCAv_end_, cm·s^−1^	9.9	0.45	7.4	0.73

*n* = 17 subjects. CV, coefficient of variation; ICC, intraclass correlation coefficient; MCAv, middle cerebral artery blood velocity; MCAv_end_, MCAv at end of exercise bout; MRT, mean response time; τ, time constant; TD, time delay.

### Associations between V̇o_2max_ and MCAv Kinetics Parameters

**Figure 3.**
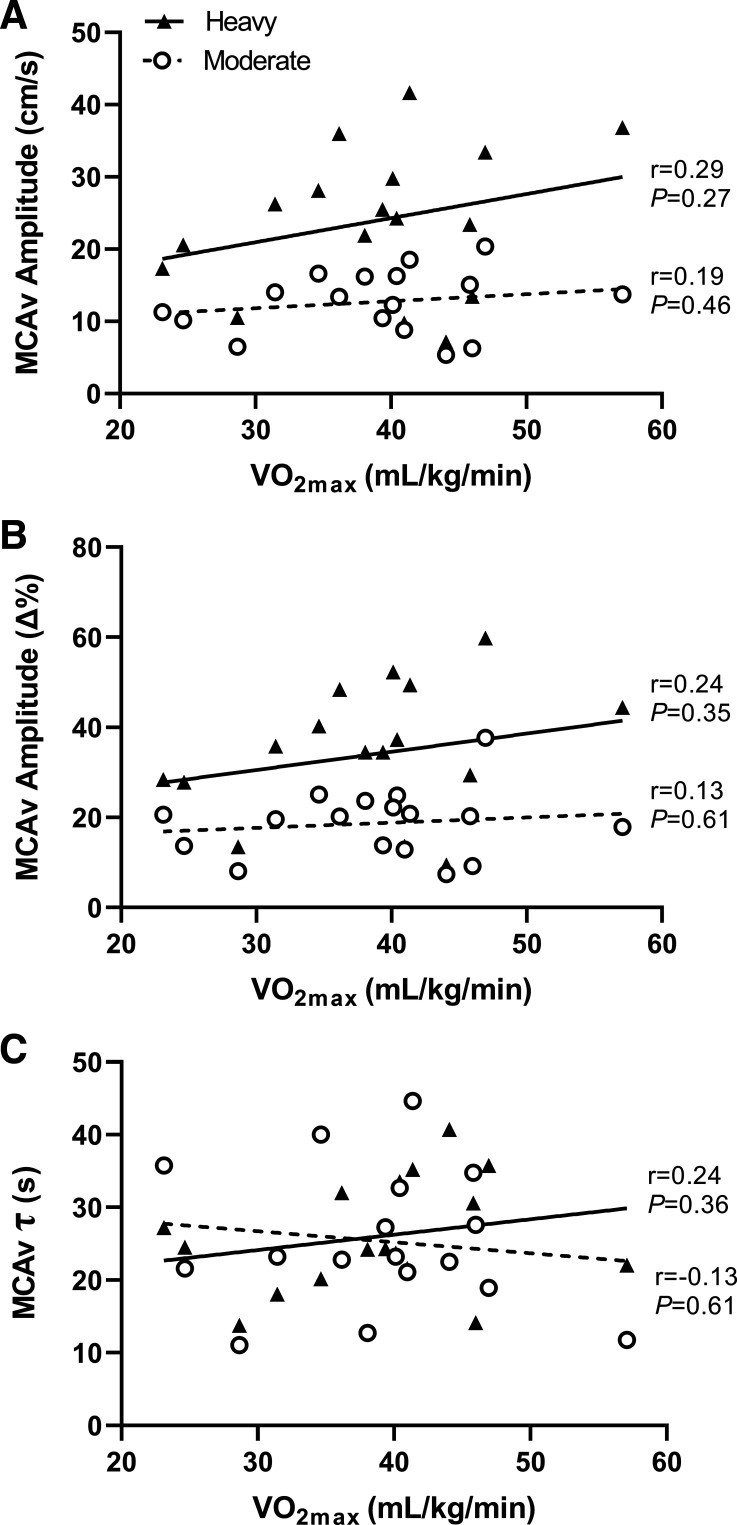
Correlations between V̇o_2max_ and MCAv amplitude (*A*: absolute terms; *B*: relative change from baseline) and MCAv time constant, τ (*C*). Data shown for moderate-intensity exercise (circles, dashed line) and heavy-intensity exercise (black triangles, solid line) (*n* = 17, 9 females). MCAv, middle cerebral artery blood velocity; V̇o_2max_, maximal oxygen uptake.

V̇o_2max_ was not significantly correlated with absolute and relative MCAv amplitude, nor with MCAv τ during moderate- and heavy-intensity exercise (Fig. 3).

### Responses of V̇o_2_, V̇e, and HR to Moderate- and Heavy-Intensity Exercise

**Figure 4.**
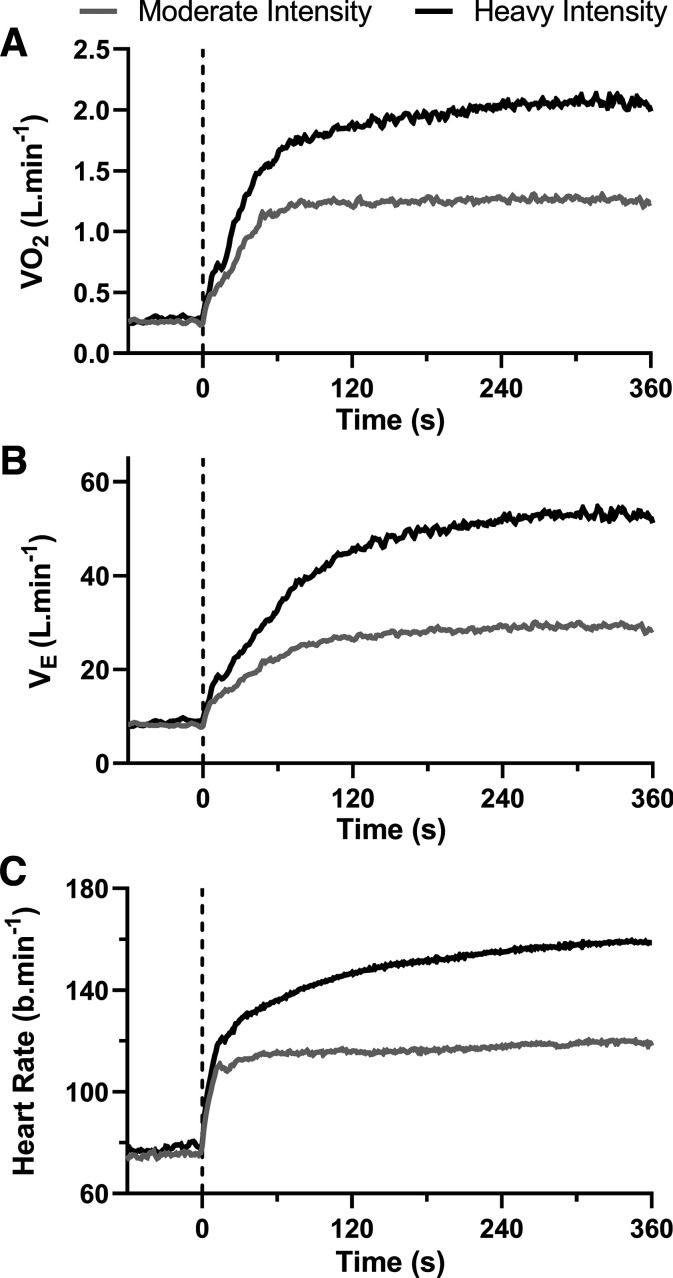
Group-averaged oxygen uptake (V̇o_2_, *A*), minute ventilation (V̇e, *B*), and heart rate responses (*C*) to moderate (gray line) and heavy (black line) intensity cycling in healthy adults (*n* = 17, 9 females).

The group-averaged responses of V̇o_2_, V̇e, and HR to moderate- and heavy-intensity exercise are shown in Fig. 4. A steady-state V̇o_2_, V̇e, and HR were achieved during moderate-intensity exercise. V̇o_2_ at the end of the exercise was 1.25 ± 0.29 L·min^−1^ and 2.06 ± 0.45 L·min^−1^, following moderate- and heavy-intensity exercise, respectively. V̇e achieved a steady state during moderate-intensity exercise, with an end-exercise value of 29.0 ± 6.2 L·min^−1^, but V̇e rose to 53.0 ± 11.3 L·min^−1^ at the end of heavy-intensity exercise. At the end of moderate-intensity exercise, a steady-state HR was reached at 121 ± 14 beats·min^−1^, but HR rose to 159 ± 11 beats·min^−1^ at the end of heavy-intensity exercise.

## DISCUSSION

This is the first study to explore the MCAv kinetic response in healthy young adults during whole body, upright cycling at a moderate and heavy intensity, completed below and above the GET, respectively. This study found that, following dynamic fluctuations in MCAv for ∼20–25 s at exercise onset, MCAv increased exponentially to moderate- and heavy-intensity cycling, and this response was well-fitted by a monoexponential model with a time delay. In line with the hypotheses, the time-based parameters of the MCAv response (τ, TD, and MRT) were not different during moderate- and heavy-intensity cycling. However, contrary to the hypothesis, the amplitude of the exponential increase in MCAv was greater during heavy-intensity exercise compared with moderate-intensity exercise. This study also observed a fall in MCAv following this initial exponential phase, which was greater during heavy-intensity exercise. However, MCAv after 6 min of exercise remained higher during heavy-intensity exercise compared with moderate-intensity exercise. This study also found that cardiorespiratory fitness was not significantly related to the MCAv kinetic response to moderate- and heavy-intensity cycling.

### Effect of Exercise Intensity on MCAv Kinetics

The present study demonstrates that the MCAv response to whole body cycling at a moderate intensity, performed below the GET, can be modeled using a monoexponential equation with a time delay. The overall MRT of the MCAv kinetic response to moderate-intensity exercise was much faster in the present study compared with previous work by Billinger et al. (2; ∼55 s vs. ∼83 s). This is likely a result of the instantaneous transition to the target work rate in the present study, with the 30 s adjustment period slowing the MCAv kinetic response in previous work (2, 6). In addition, appropriate test-retest reproducibility of the MCAv kinetic response was observed in the present study, with the coefficient of variation for MCAv kinetics parameters (baseline, amplitude, and MRT) similar to Billinger et al. (2). These levels of reproducibility are also in-line with, or smaller than, kinetics parameters of MCAv to CO_2_ breathing, and typical for studies utilizing kinetics analyses (27).

Contrary to the work by Billinger et al. (2), the present study found that, following an exponential increase at exercise onset, MCAv did not maintain a clear steady state during moderate-intensity cycling. In the majority of cases, a fall in MCAv was observed after an initial exponential rise. This observation is particularly interesting given that, below the GET, steady-state responses are observed for V̇o_2_, V̇co_2_, V̇e, and heart rate (1, 7). These data suggest that MCAv responds differently to other variables when exercising below the GET, and may have its own unique threshold for steady-state responses, which has yet to be explored. Future research should investigate potential reasons underpinning the absence of steady-state MCAv in these participants, with potentially important interactions between the respiratory system (12), hyperventilation (15), and $\text{P}\text{E}{\text{T}}_{\text{C}{\text{O}}_{2}}$ (14) during exercise.

The effect of exercise intensity on MCAv kinetics has only been explored once before, with Witte et al. (6) observing a greater MCAv amplitude during “moderate” intensity stepper exercise compared with “low” intensity stepper exercise. This supports evidence from incremental exercise, where increases in CBF are greatest during moderate-intensity exercise compared with both low- and high-intensity exercise (13, 14). However, Witte et al. (6) found that the time-based kinetics parameters (τ, TD, and MRT) of the MCAv response to low- and moderate-intensity stepping exercises were not different between intensities. The present study is the first to explore the MCAv kinetic response to heavy-intensity exercise, performed above the GET, and also found that exercise intensity did not alter the time-based kinetics parameters of the MCAv response to exercise. However, heavy-intensity exercise elicited an initial exponential increase in MCAv around twice that observed during moderate-intensity exercise (∼34% vs. ∼19%), which disagrees with the common (mis)interpretation from incremental data. This is an important finding and highlights that evidence on MCAv responses to incremental exercise cannot be translated into constant work-rate exercise.

As the 6-min exercise bout progressed, MCAv decreased by a greater magnitude during heavy-intensity exercise compared with moderate-intensity exercise. This is likely due to the influence of a greater degree of hyperventilation-induced hypocapnia when exercising above the GET, resulting in cerebral vasoconstriction and reductions in MCAv (14, 15). Nevertheless, at the end of exercise, MCAv remained significantly elevated above baseline during heavy-intensity exercise compared with moderate-intensity exercise. Given the exercise bout was terminated at 6 min, future research should explore if MCAv returns to levels near, or below, the baseline at exhaustion during constant work-rate exercise as is observed during incremental exercise (14). The cerebrovascular responses to high-intensity exercise have not been widely investigated, and the present study provides novel data that contributes to a growing discussion surrounding high-intensity exercise and the brain (13, 28).

A striking feature of the MCAv response to exercise of both intensities in the present study was the marked, dynamic, and consistent fluctuation seen in MCAv before the exponential-like increase, which lasted for ∼20–25 s. This novel observation, not described in previous research, is likely a consequence of an instantaneous transition from rest to exercise, as opposed to the 30 s adjustment period used in previous research (2) and appears to not be intensity-dependent. During this period, dynamic changes in blood pressure and $\text{P}\text{E}{\text{T}}_{\text{C}{\text{O}}_{2}}$ are likely occurring, which are important regulatory factors of MCAv (29). In particular, cerebral autoregulation takes ∼5 s to protect against sudden changes in blood pressure (30, 31), and a delay is also present in the cerebrovascular response to changes in $\text{P}\text{E}{\text{T}}_{\text{C}{\text{O}}_{2}}$ (32), which may be contributing to these dynamic fluctuations in MCAv seen at exercise onset. Whether this dynamic MCAv onset profile is altered in populations with compromised cerebral autoregulation or cerebrovascular reactivity, and the implications on cerebral hemodynamics, remains to be explored. This would be an important consideration, where the transition from rest to exercise may elevate the risk of sudden hyper-perfusion injury in some groups (13, 28).

### Relationship between Cardiorespiratory Fitness and MCAv Kinetics

Witte et al. (6) found a significant, positive correlation between estimated V̇o_2max_ and MCAv amplitude during both low- and moderate-intensity exercise. Using cardiopulmonary exercise testing and a direct measurement of V̇o_2max_ (rather than questionnaire; 33), the present study extends these findings and is the first to report no significant association between V̇o_2max_ and MCAv kinetics (amplitude and τ) in healthy young adults during moderate- or heavy-intensity exercise. The influence of fitness on cerebral hemodynamics remains unclear, however, a recent systematic review found that higher cardiorespiratory fitness was associated with increased cerebrovascular reactivity to CO_2_ (CVR; 34), which could translate into a greater MCAv amplitude during exercise. This has been suggested to be related to improved endothelial function and cerebral angiogenesis in individuals with higher levels of cardiorespiratory fitness, which results in elevated levels of CBF (35). However, some data suggests no relationship between V̇o_2max_ and CVR (36), and inferences are likely influenced by disparities in measurement technique (TCD vs. MRI) and stimulus used (34). Nevertheless, the present results suggest that any potential fitness-related elevations in CVR at rest do not translate to an increased MCAv amplitude during moderate- and heavy-intensity cycling in young, healthy adults (V̇o_2max_ range: 23–55 mL·kg·min^−1^).

### Study Considerations

The present study builds on previous work, which used predicted HRmax to prescribe exercise intensity (2–6) by exploring the MCAv kinetic response to upright cycling above and below the GET, utilizing an instantaneous transition to the target work-rate. In addition, the present study used repeat transitions (3 bouts of each intensity on separate days) to enhance the signal-to-noise ratio of acquired data, and observed appropriate levels of reproducibility for kinetics parameters during both moderate- and heavy-intensity exercise.

The present study used transcranial Doppler (TCD) ultrasound to measure MCAv, allowing continuous, noninvasive measurement with high temporal resolution during whole body exercise for kinetic analyses (2). However, TCD does not measure MCA diameter, and cerebral blood velocity is only an appropriate surrogate of CBF if vessel diameter remains constant. Although changes in MCA diameter may occur during steady state increases in $\text{P}\text{E}{\text{T}}_{\text{C}{\text{O}}_{2}}$ of as little as 4.5 mmHg (37), TCD is considered an appropriate and practical tool to estimate CBF during exercise (15) and has been utilized in other work investigating MCAv kinetics during exercise in adults (2, 3, 6).

The present study was unable to explore regional differences in CBF during exercise, due to the measurement of bilateral MCAv. Regional differences in CBF during incremental exercise have been observed, with continuous elevations in blood flow in posterior arteries [vertebral and posterior cerebral (PCA) arteries] with increasing exercise intensity, contrary to the bi-phasic MCAv response (14, 38). However, during a single, 30-s bout of high-intensity constant work-rate exercise, no regional differences were found between the MCAv and PCAv responses in healthy females (39). Nevertheless, further investigation of the intensity-dependent and region-specific CBF responses during constant work-rate, compared with incremental, exercise are needed.

An additional consideration is that the menstrual cycle was not controlled for in the female participants. Whether or not the menstrual cycle should be controlled for in cardiovascular research has been debated (40, 41). Recent research found no effect of menstrual cycle phase on cerebral autoregulation in heathy, young females (42, 43), but no data are available on the effect of menstrual cycle on cerebrovascular responses to exercise. In addition, the test-retest reproducibility of MCAv kinetics outcomes was appropriate and similar to previous work where female participants were only tested in the early follicular phase (2), suggesting differences in the menstrual cycle phase may not be confounding the study’s main findings.

In addition, all participants were young and healthy, and these findings cannot be extrapolated into other groups, such as older adults, where known kinetic differences during moderate-intensity exercise exist (3). Given that this is the first study to explore the MCAv kinetic response to whole body cycling, future research is needed to establish “normal” kinetic profiles, and how these are altered by factors such as age, disease, training, and environmental factors. Finally, the present study did not collect measurements of $\text{P}\text{E}{\text{T}}_{\text{C}{\text{O}}_{2}}$ and blood pressure during exercise, which are important regulators of CBF (14, 29). Future research exploring the kinetic responses of these regulatory factors to exercise will further elucidate the physiological mechanisms underpinning the intensity-dependent MCAv kinetic response to exercise in adults, and how this can be influenced by important factors such as age (3), maturation (44, 45), disease status (4, 5), and exercise intensity (6).

### Conclusions

This is the first study to kinetically model the MCAv response to moderate- and heavy-intensity cycling in healthy adults, and found the time-based parameters (τ, TD, and MRT) of MCAv were not different between both intensities. This study found that the amplitude of the exponential increase in MCAv to heavy-intensity exercise was greater compared with moderate-intensity exercise, but the fall in MCAv during the bout was also greater during heavy-intensity exercise. Nevertheless, MCAv at the end of the exercise bout remained elevated in heavy-intensity exercise compared with moderate-intensity exercise. Finally, cardiorespiratory fitness was not associated with the MCAv kinetic response to moderate nor heavy-intensity exercise in healthy adults. These novel analysis techniques form an important area for future research to further explore cerebrovascular responses and regulation during exercise.

## GRANTS

This work was supported by the QUEX Institute (University of Queensland and University of Exeter).

## DISCLOSURES

No conflicts of interest, financial or otherwise, are declared by the authors.

## AUTHOR CONTRIBUTIONS

M.E.W., A.R.B., J.S.C., T.G.B., and B.B. conceived and designed research; M.E.W. performed experiments; M.E.W., A.R.B., O.W.T., and B.B. analyzed data; M.E.W., A.R.B., O.W.T., and B.B. interpreted results of experiments; M.E.W. prepared figures; M.E.W. drafted manuscript; M.E.W., A.R.B., O.W.T., J.S.C., T.G.B., and B.B. edited and revised manuscript; M.E.W., A.R.B., O.W.T., J.S.C., T.G.B., and B.B. approved final version of manuscript.
